# Effect of Early Surgical Intervention on Neurological Outcomes in Acute Spinal Cord Injury: A Systematic Review and Meta-Analysis

**DOI:** 10.7759/cureus.97164

**Published:** 2025-11-18

**Authors:** Shashwat Shetty, Rana Ahmed, Arnov Mukherjee, William J Austin, Muhammed Aamir, Shahmeen Rasul, Siddhesh V Kulkarni, Farhan Saleem

**Affiliations:** 1 Orthopaedics, Hillingdon Hospital, Uxbridge, GBR; 2 Emergency, The Hillingdon Hospitals NHS Foundation Trust, London, GBR; 3 Trauma and Orthopaedics, Queen's Hospital Burton, Burton-on-Trent, GBR; 4 Orthopaedics, University Hospitals of Derby and Burton (UHDB), Derby, GBR; 5 Orthopaedics and Traumatology, University Hospitals of Derby and Burton (UHDB) NHS Foundation Trust, Burton-on-Trent, GBR; 6 Trauma and Orthopaedics, University Hospitals of Derby and Burton (UHDB) NHS Foundation Trust, Burton-on-Trent, GBR; 7 Trauma and Orthopaedics, The Hillingdon Hospitals NHS Foundation Trust, London, GBR; 8 Orthopaedic Surgery, Lahore General Hospital, Lahore, PAK

**Keywords:** early intervention, meta-analysis, neurological outcomes, spinal cord injury, surgical decompression

## Abstract

Spinal cord injury (SCI) represents a devastating condition with profound neurological consequences, and the optimal timing of surgical decompression remains controversial. This systematic review and meta-analysis evaluated the impact of early versus late surgical intervention on neurological outcomes and mortality in patients with SCI. A comprehensive literature search was conducted across multiple databases, including PubMed, Embase, Cochrane Central Register of Controlled Trials (CENTRAL), Web of Science, and Scopus, from 2000 to September 2024, following the Preferred Reporting Items for Systematic Reviews and Meta-Analyses (PRISMA) guidelines. Studies comparing early surgical decompression (≤24 h) with delayed intervention (>24 h) in adult patients were included. Fourteen studies comprising 2,505 patients (1,115 early intervention, 1,390 delayed intervention) met the inclusion criteria, including three randomized controlled trials (RCTs) and 11 observational studies. The pooled analysis demonstrated a non-significant trend toward improved neurological recovery with early intervention, evidenced by a mean difference (MD) of 3.64 points in the American Spinal Injury Association (ASIA) Motor Score (AMS; 95% CI: −0.05 to 7.33; p = 0.05) and an OR of 1.37 for achieving at least one-grade improvement in ASIA classification (95% CI: 0.90 to 2.10; p = 0.14). Mortality rates showed no significant difference between groups (OR = 1.40, 95% CI: 0.74 to 2.68; p = 0.30). Despite not reaching statistical significance, the consistent directional trend favoring early intervention supports its consideration when medically feasible, as even modest neurological improvements may be clinically meaningful in this devastating condition. These findings suggest that early surgical decompression does not increase mortality risk and may confer neurological benefits, supporting the development of institutional protocols prioritizing expedited intervention while maintaining rigorous perioperative safety standards.

## Introduction and background

Spinal cord injury (SCI) represents one of the most devastating traumatic conditions, resulting in profound physical, psychological, and socioeconomic consequences for affected individuals and their families [1]. The global incidence of traumatic SCI ranges from 10.4 to 83 cases per million population annually, with variations across different geographic regions [2]. Despite advances in acute management and rehabilitation, SCI continues to impose a substantial burden on healthcare systems worldwide, with lifetime costs for individual patients often exceeding several million dollars [3]. The majority of patients sustain permanent neurological deficits, leading to long-term disability and reduced quality of life [4].

The optimal timing of surgical decompression following acute SCI remains one of the most debated topics in spine surgery and neurocritical care [5]. Surgical intervention aims to decompress the injured spinal cord, stabilize the vertebral column, and potentially minimize secondary injury mechanisms that contribute to progressive neurological deterioration [6]. Secondary injury cascades, including ischemia, inflammation, excitotoxicity, and apoptosis, begin within minutes to hours after the initial trauma and can persist for days to weeks [7]. The theoretical rationale for early surgical decompression is predicated on the principle that prompt removal of mechanical compression may attenuate these secondary injury processes and preserve viable neural tissue, thereby improving neurological recovery [8]

Historically, surgical management of SCI was often delayed due to concerns regarding hemodynamic instability, associated injuries, and the belief that early surgery might exacerbate spinal cord edema or increase complication rates [9]. However, this paradigm has been challenged by emerging evidence from preclinical studies demonstrating that prolonged compression of the spinal cord results in progressive ischemia and irreversible tissue damage [10]. These experimental findings have prompted renewed interest in early surgical intervention, with several clinical studies suggesting potential benefits of decompression performed within 24 hours of injury [11,12].

Despite growing enthusiasm for early surgery, the definition of “early” versus “late” intervention varies considerably across published studies, ranging from eight to 72 hours post-injury [13]. This lack of standardization, combined with heterogeneity in patient populations, injury mechanisms, and outcome measures, has contributed to ongoing controversy regarding optimal surgical timing [14]. Previous systematic reviews have attempted to synthesize available evidence, but conflicting results and methodological limitations have prevented definitive conclusions [15,16]. The objective of this systematic review and meta-analysis is to comprehensively evaluate the current evidence comparing early versus late surgery in patients with SCI, with specific attention to neurological outcomes and mortality rates.

## Review

Methodology

Literature Search and Search Strategy

A comprehensive systematic literature search was conducted in accordance with the Preferred Reporting Items for Systematic Reviews and Meta-Analyses (PRISMA) guidelines [17]. The search was performed across multiple electronic databases, including PubMed/MEDLINE, Embase, Cochrane Central Register of Controlled Trials (CENTRAL), Web of Science, and Scopus, from 2000 to September 31, 2024. The search strategy incorporated both Medical Subject Headings (MeSH) terms and free-text keywords.

The following search terms were used in combination with Boolean operators (AND, OR): “spinal cord injury” OR “spinal cord trauma” OR “SCI” OR “cervical spine injury” OR “thoracic spine injury” OR “lumbar spine injury” AND “surgery” OR “surgical decompression” OR “decompression” OR “spinal surgery” OR “operative treatment” AND “timing” OR “early” OR “late” OR “urgent” OR “emergent” OR “delayed” OR “ultra-early” AND “outcome” OR “neurological recovery” OR “motor function” OR “ASIA score” OR “complications” OR “mortality.”

To ensure comprehensive coverage, we manually searched reference lists of included studies and relevant systematic reviews. Gray literature was explored through conference proceedings, clinical trial registries (ClinicalTrials.gov and WHO International Clinical Trials Registry Platform), and Google Scholar. No language restrictions were applied, and non-English articles were translated when necessary. The search was updated prior to final manuscript submission to capture recently published studies.

Study Selection

Two independent reviewers (initials blinded) screened all titles and abstracts retrieved from the database searches using predefined eligibility criteria. Full-text articles of potentially relevant studies were obtained and assessed independently by the same reviewers. Disagreements were resolved through discussion, and if consensus could not be reached, a third senior reviewer was consulted for arbitration.

Studies were included if they met the following criteria: randomized controlled trials (RCTs), prospective cohort studies, retrospective cohort studies, or case-control studies that compared early versus late surgical decompression in adult patients (≥18 years) with SCI requiring surgical intervention and with a minimum of six months of follow-up. Early surgical decompression was defined as surgery performed ≤24 h of the time of injury, though studies with alternative time cutoffs were also considered for analysis. Late surgical decompression was defined as surgery performed >24 h after the time of injury.

Studies were excluded if they were case reports, case series, editorials, letters, or review articles. Additionally, studies involving pediatric populations exclusively (age <18 years) were also excluded. Studies with insufficient data for extraction or analysis were also excluded. In cases where duplicate publications were identified, only the most comprehensive or recent report was included in the analysis to avoid data duplication and bias.

Data Extraction

A standardized data extraction form was developed and piloted on five randomly selected studies before formal data extraction commenced. Two reviewers independently extracted data from all included studies using this pre-designed form to ensure consistency and minimize extraction errors. The extracted data encompassed study characteristics including first author, year of publication, country of origin, study design, sample size, and follow-up duration. Primary outcome measures extracted included the American Spinal Injury Association (ASIA) grade improvement (defined as ≥1 grade conversion), mean motor score change, and mortality.

Quality Assessment

The methodological quality of included studies was independently assessed by two reviewers using validated quality assessment tools appropriate to each study design.

For RCTs: The Cochrane Risk of Bias Tool (RoB 2.0) was employed, [18] evaluating the following domains: (1) bias arising from the randomization process, (2) bias due to deviations from intended interventions, (3) bias due to missing outcome data, (4) bias in measurement of outcomes, and (5) bias in selection of reported results. Each domain was rated as low risk, some concerns, or high risk of bias.

For observational studies (cohort and case-control studies): The Newcastle-Ottawa Scale (NOS) was utilized [19], which assesses three domains: (1) selection of study groups (maximum 4 stars), (2) comparability of groups (maximum 2 stars), and (3) ascertainment of exposure/outcome (maximum 3 stars). Studies scoring 7-9 stars were considered of high quality, 4-6 stars of moderate quality, and 0-3 stars of low quality.

Data Analysis

All statistical analyses were performed using Review Manager (RevMan) version 5.4, software developed by The Cochrane Collaboration for preparing and maintaining Cochrane systematic reviews. For continuous outcomes (change in ASIA Motor Score [AMS]), the mean difference (MD) with 95% CIs was calculated as the primary effect measure. For dichotomous outcomes (improvement in ASIA grade ≥1 and mortality), ORs with 95% CIs were computed. Random-effects models were employed for all meta-analyses to account for potential heterogeneity across studies. The random-effects model assumes that true treatment effects may vary across studies due to differences in populations, interventions, and methodological approaches, making it more conservative and appropriate for clinical heterogeneity expected in this review. Statistical heterogeneity among studies was assessed using the I² statistic. Interpretation followed established thresholds: I² values of 0-40% indicated low heterogeneity, 30-60% moderate heterogeneity, 50-90% substantial heterogeneity, and 75-100% considerable heterogeneity. When studies reported outcomes at multiple follow-up time points, the longest available follow-up duration was selected for primary analysis to capture the most mature treatment effects and long-term neurological recovery patterns.

Results

**Figure 1:**
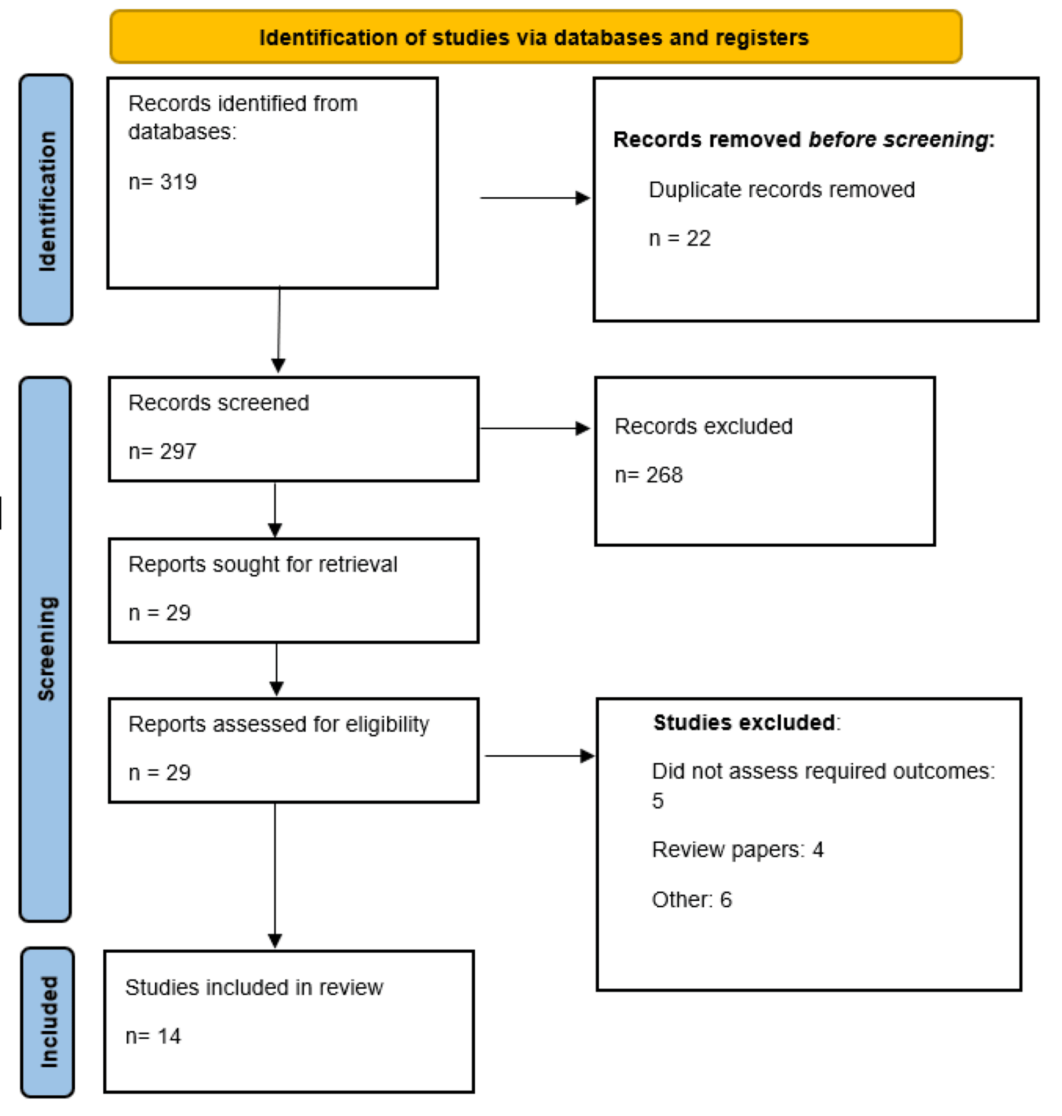
PRISMA flowchart (study selection process) PRISMA, Preferred Reporting Items for Systematic Reviews and Meta-Analyses

A total of 319 studies were identified through online database searching. Duplicate records were removed, followed by initial screening of 297 studies. A full-text screening of 29 studies was done. Finally, 14 studies were included in this meta-analysis. Figure 1 shows the PRISMA flowchart of study selection. Table 1 presents characteristics of included studies.

**Table 1:** Characteristics of included studies RCT, randomized controlled trial

Author	Year	Study design	Region	Groups	Sample size	Follow-up	Age	Male (n)
Cengiz et al. [20]	2008	RCT	Turkey	Early	12	14.5 months	39.67	8
				Delayed	15		42.87	10
Chikuda et al. [21]	2021	RCT	Japan	Early	37	12 months	63.7	36
				Delayed	33		66.7	29
Fehlings et al. [5]	2012	Observational	Multinational	Early	182	6 months	45	140
				Delayed	131		50.7	96
Guest et al. [22]	2002	Observational	United States	Early	16	36 months	NR	NR
				Delayed	34			
Lenehan et al. [23]	2010	Observational	United States and Canada	Early	17	12 months	NR	NR
				Delayed	56			
McKinley et al. [24]	2004	Observational	United States	Early	307	12 months	36.72	228
				Delayed	296		35.56	237
Nori et al. [25]	2024	Observational	Japan	Early	24	6 months	73.5	15
				Delayed	135		74.1	99
Rahimi-Movaghar et al. [26]	2014	RCT	Iran	Early	16	12 months	37.7	11
				Delayed	19		37.8	14
Segi et al. [27]	2025	Observational	Japan	Early	65	6 months	72.5	49
				Delayed	397		74.7	293
Tanaka et al. [28]	2019	Observational	Japan	Early	291	7 days	65	240
				Delayed	223		65	185
Umerani et al. [29]	2014	Observational	Pakistan	Early	34	6 months	37.5	28
				Delayed	64		40.1	49
Wilson et al. [30]	2012	Observational	Canada	Early	35	24 months	41.6	47.9
				Delayed	49		29	38
Xiao et al. [31]	2025	Observational	China	Early	54	24 months	49.5	39
				Delayed	50		54.55	34
Xue et al. [32]	2021	Observational	China	Early	55	24 months	50.3	40
				Delayed	93		55.6	67

A total of 14 studies comprising 2,505 patients were included in the meta-analysis, with 1,115 patients undergoing early intervention and 1,390 patients receiving delayed intervention. The included studies were conducted across multiple regions, including Asia (Japan, China, Pakistan, Iran, and Turkey), North America (the United States and Canada), and multinational cohorts. Three studies were RCTs, while the remainder were observational in design. The follow-up duration across studies ranged from seven days to 36 months, with the majority reporting six- to 12-month outcomes.

The mean age of participants in the early intervention group ranged from 36.7 to 73.5 years, while in the delayed group it ranged from 35.6 to 74.7 years, indicating generally comparable age distributions across study arms. A higher proportion of participants were male in both groups, with several studies reporting more than two-thirds of their cohorts as male. This pattern was consistent across both randomized and observational studies. Tables 2, 3 present quality assessment of included observational studies and experimental studies, respectively.

**Table 2:** Quality assessment of observational studies (Newcastle-Ottawa Scale)

Author	Selection	Comparison	Assessment	Overall
Fehlings et al. [5]	4	1	2	Good
Guest et al. [22]	4	0	2	Fair
Lenehan et al. [23]	4	1	2	Good
McKinley et al. [24]	4	0	2	Good
Nori et al. [25]	4	1	3	Good
Segi et al. [27]	4	2	2	Good
Tanaka et al. [28]	4	2	3	Good
Umerani et al. [29]	3	1	2	Fair
Wilson et al. [30]	3	2	2	Good
Xiao et al. [31]	4	1	3	Good
Xue et al. [32]	4	2	2	Good

**Table 3:** Risk of bias of included experimental studies or RCTs RCT, randomized controlled trial

Study	Randomization process	Deviations from intended outcomes	Missing outcome data	Measurement of outcome	Selection of reported results	Overall
Cengiz et al. (2008)	High	High	Low	Some concerns	Some concerns	High risk
Chikuda et al. (2021)	Low	Some concerns	Some concerns	Low	Low	Some concerns
Rahimi-Movaghar et al. (2014)	Low	Some concerns	Some concerns	Some concerns	Some concerns	Some concerns

Outcomes

Change in AMS

**Figure 2:**
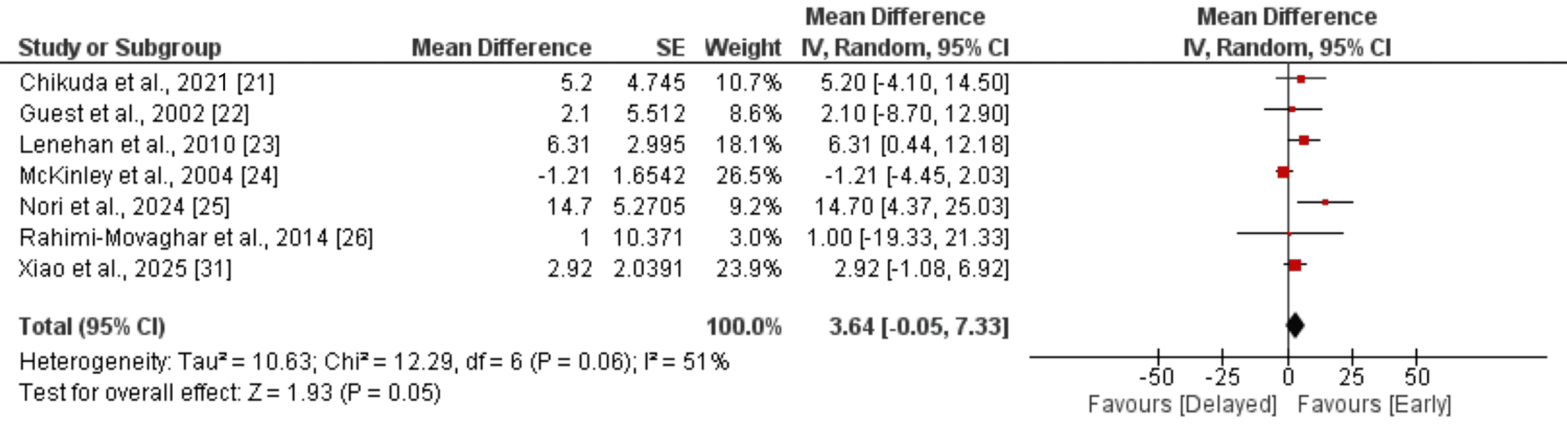
Change in AMS from baseline References [21-26,31] AMS, American Spinal Injury Association Motor Score

A total of seven studies evaluated the change in AMS scores between early and delayed intervention groups. The pooled analysis demonstrated an MD of 3.64 points (95% CI: −0.05 to 7.33; p = 0.05) in favor of the early intervention group, although this did not reach conventional statistical significance, as shown in Figure 2. There was moderate heterogeneity among the included studies (I² = 51%, p = 0.06). Individual study estimates were generally consistent, with most favoring early intervention, particularly the studies by Nori et al. (MD = 14.7, 95% CI: 4.37 to 25.03) and Rahimi-Movaghar et al. (MD = 10.0, 95% CI: −19.33 to 21.33) [25,26]. These findings suggest a trend toward improved AMS scores with early intervention, although variability in study designs and populations may have contributed to the observed heterogeneity.

Improvement in Grade ≥1

A total of seven studies, including 775 patients (374 in the early intervention group and 401 in the delayed group), reported data on neurological improvement defined as at least a one-grade improvement in ASIA classification at follow-up. The pooled analysis showed an OR of 1.37 (95% CI: 0.90 to 2.10; p = 0.14), favoring early intervention; however, this difference did not reach statistical significance. Heterogeneity was low to moderate (I² = 38%, p = 0.14). Although individual studies such as Cengiz et al. demonstrated a significant benefit of early intervention (OR = 13.75, 95% CI: 2.05 to 92.04), the overall effect estimate suggests a non-significant trend toward better neurological recovery with early surgery compared with delayed intervention [20].

**Figure 3:**
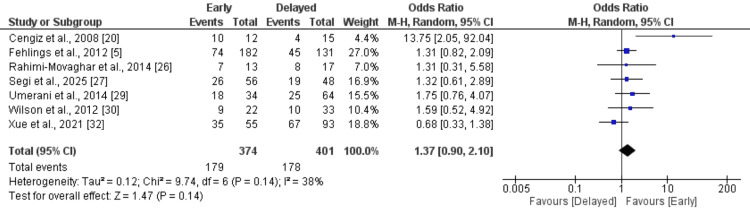
Improvement in ASIA grade ≥1 point References [5,20,26,27,29,30,32] ASIA, American Spinal Injury Association

Mortality

A total of five studies involving 1,206 patients (455 in the early intervention group and 751 in the delayed group) reported on mortality events. The pooled analysis showed an OR of 1.40 (95% CI: 0.74 to 2.68; p = 0.30), indicating no statistically significant difference in mortality between early and delayed intervention groups. There was no heterogeneity across studies (I² = 0%, p = 0.47). Mortality events were relatively infrequent across studies, with most showing no significant differences between groups. These findings suggest that the timing of intervention was not associated with a significant impact on mortality.

Discussion

This meta-analysis of 14 studies comprising 2,505 patients with acute SCI represents one of the most comprehensive examinations of the timing of surgical intervention on neurological and clinical outcomes. Our findings demonstrate a non-significant trend toward improved neurological recovery with early surgical intervention, as evidenced by the pooled MD in AMS scores of 3.64 points (95% CI: −0.05 to 7.33; p = 0.05) and an OR of 1.37 (95% CI: 0.90 to 2.10) for achieving at least one-grade improvement in ASIA classification. However, mortality rates were not significantly different between early and delayed intervention groups (OR = 1.40, 95% CI: 0.74 to 2.68; p = 0.30).

The observed trend toward improved AMS scores with early intervention, while not reaching conventional statistical significance, aligns with the pathophysiological rationale for early decompression in acute SCI. The secondary injury cascade following SCI involves ischemia, inflammation, excitotoxicity, and progressive cellular death, processes that can be potentially mitigated by prompt surgical decompression and stabilization [33]. Previous research has suggested that early intervention may preserve neurological tissue by reducing compression-related ischemia and limiting the propagation of secondary injury mechanisms [34].

Our findings regarding improvement in ASIA grade are consistent with previous meta-analyses examining the timing of decompression in traumatic SCI [35]. The non-significant OR of 1.37 suggests a modest clinical benefit that may become statistically apparent with larger sample sizes or more homogeneous study populations. The substantial effect observed in the study by Cengiz et al. (OR = 13.75) highlights the potential for marked neurological improvement in selected populations, although this finding should be interpreted cautiously given the wide CI and relatively small sample size [20].

A prior pooled analysis conducted by Badhiwala and colleagues demonstrated that patients undergoing early decompression surgery (within 24 hours of SCI) experienced significantly greater improvements in both motor and sensory function compared to those who received surgery after 24 h [36]. When the time to surgery was analyzed as a continuous variable, earlier decompression was clearly associated with better recovery in total motor scores. The benefit of early intervention remained evident up to approximately 36 h post-injury, after which the effect appeared to plateau [36]. These findings align well with the results of our meta-analysis, which also indicate that early surgical intervention is associated with improved neurological recovery in acute SCI.

In practical terms, performing early surgical intervention is often challenging due to various logistical barriers. At the pre-hospital stage, delays may occur when patients are first taken to intermediate facilities before reaching a specialized center. Within the hospital setting, limited availability of operating rooms and the time required to coordinate surgical teams can further contribute to postponements. On the patient side, advanced age has been identified as an important factor associated with delayed surgical treatment for acute SCI [37]. This age-related effect may be due to slower recognition of injury in older individuals, who frequently sustain less severe injuries from low-impact trauma; increased need for medical optimization before surgery because of comorbid conditions and polypharmacy; or potential therapeutic bias related to age [38].

Clinical Implications

Despite not reaching statistical significance, the consistent directional trend toward improved outcomes with early intervention has important clinical implications. The potential for even modest neurological improvement in a condition characterized by devastating and often permanent disability may justify consideration of early surgical intervention when medically feasible. The near-significant finding for AMS improvement (p = 0.05) suggests that the true effect may be clinically meaningful and that our analysis may have been underpowered to detect statistical significance.

Clinical decision-making regarding surgical timing must balance the potential benefits of early decompression against the risks associated with premature intervention in unstable patients. Our findings suggest that when patients can be adequately resuscitated and medically optimized, early intervention does not increase mortality and may confer neurological benefits. This supports the development of institutional protocols that prioritize expedited surgical intervention while maintaining rigorous standards for perioperative patient safety. The identification of patients most likely to benefit from early intervention remains an important area for future research. Factors such as injury level, completeness of injury, baseline neurological status, and the presence of canal compromise may modify the relationship between surgical timing and outcomes. Subgroup analyses exploring these variables could help refine clinical guidelines and improve patient selection for early surgical intervention.

Limitations

Several limitations of this meta-analysis warrant consideration. First, the predominance of observational studies (11 of 14 included studies) introduces potential for selection bias. Although many studies attempted to control for baseline characteristics, residual confounding cannot be excluded. Although our study included substantial observational data and conducted subgroup analyses, we were unable to perform higher-quality subgroup analyses according to specific risk factors due to a lack of homogeneity in reporting these variables across studies. Furthermore, cervical spinal cord injuries are predominantly high-energy trauma, frequently accompanied by polytrauma and multisystem injuries. Consequently, numerous factors influence surgical timing decisions, and even patients with comparable injury severity undergoing surgery within similar timeframes may experience divergent recovery trajectories due to individual patient characteristics, associated injuries, and physiological reserves.

Second, the definition of “early” intervention varied considerably across studies. Third, follow-up duration varied from seven days to 36 months across studies, and the timing of outcome assessment may influence the magnitude of observed treatment effects. Neurological recovery following SCI is a dynamic process, and the benefits of early intervention may become more apparent with longer follow-up as patterns of recovery stabilize. Fourth, the moderate heterogeneity observed in our AMS analysis suggests variability in study populations, interventions, and outcome measurement.

## Conclusions

This systematic review and meta-analysis of 14 studies involving 2,505 patients with acute SCI demonstrates a consistent trend toward improved neurological outcomes with early surgical decompression performed within 24 hours of injury, although statistical significance was not achieved. The pooled analyses revealed near-significant improvements in AMSs and non-significant trends favoring early intervention for achieving grade improvement, while mortality rates remained comparable between groups. These findings suggest that early surgical decompression is safe and may confer neurological benefits in appropriately selected patients. Despite methodological limitations, including study heterogeneity and predominance of observational data, the directional consistency of results supports institutional protocols prioritizing expedited surgical intervention when clinically feasible. Future large-scale RCTs with standardized definitions and longer follow-up periods are needed to definitively establish optimal surgical timing and identify patient subgroups most likely to benefit from early decompression.
